# *Planes* on a Snake? On the Identities of Crab Larvae Rafting on Sea Snakes

**DOI:** 10.1002/ece3.73742

**Published:** 2026-05-31

**Authors:** Soma Elefánti, Robert Lasley, Gustav Paulay, Joseph B. Pfaller

**Affiliations:** ^1^ Florida Museum of Natural History University of Florida Gainesville Florida USA; ^2^ Guam Center for Biodiversity Research, Invertebrate Zoology University of Guam Mangilao USA; ^3^ Archie Carr Center for Sea Turtle Research and Department of Biology University of Florida Gainesville Florida USA

**Keywords:** epibionts, Grapsidae, *Hydrophis platurus*, megalopae, species delimitation

## Abstract

Rafting on surface‐drifting flotsam or pelagic animals may represent an important behavioral strategy for larval transport and recruitment among brachyuran crabs. For some, like crabs of the genus *Planes* (Grapsidae), rafting has even become an adult lifestyle. After a 2012 study reported high numbers of unidentified grapsid megalopae rafting on pelagic sea snakes ( *Hydrophis platurus* ) off Pacific Costa Rica, we hypothesized their identity to be *Planes minutus* , which are commonly found rafting on flotsam and sea turtles in the area. To test the “*Planes* on a snake” hypothesis, we sequenced barcoding genes (CO1 and/or 16S) for 106 individual megalopae collected from sea snakes and compared with those of known species in the region. Three distinct species were detected, and all were in the predominately inter‐ to supratidal family Grapsidae: *Pachygrapsus socius*, *Goniopsis pulchra*, and *Grapsus grapsus* . No *Planes* were detected. While the “*Planes* on a snake” hypothesis was refuted, our results suggest that larval rafting may be a successful strategy for recruitment in inter‐ and supratidal grapsid crabs, but not other inter‐ to supratidal crabs nor *Planes*. The prevalence of larval rafting only in grapsid crabs suggests that the behavior may have provided the necessary precursor for the evolution of an obligate rafting species, like *Planes*.

## Graphical abstract

We sequenced COI and 16S rRNA genes from 106 crab megalopae found rafting on the pelagic sea snake *Hydrophis platurus* to test whether they were the obligate rafting species *Planes minutus* . Instead, all larvae belonged to three inter‐ to supratidal grapsid crabs—*Pachygrapsus socius*, *Goniopsis pulchra*, and *Grapsus grapsus* —revealing no evidence of *Planes*. These results show that larval rafting on sea snakes is common in grapsid shore crabs and may represent a lineage‐specific precursor that enabled the evolution of obligate rafting in *Planes*.

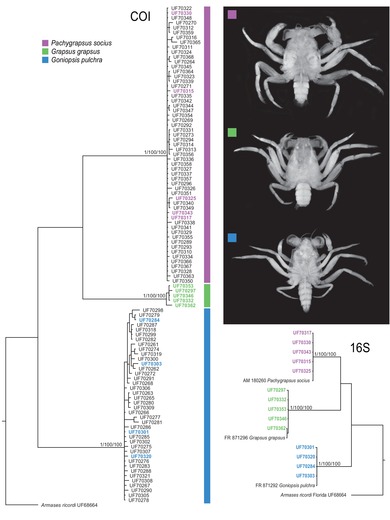

## 1 Introduction

For marine brachyuran crabs, the megalopal larval stage represents a key transition between the pelagic habitats of planktonic larvae and the benthic, intertidal, or terrestrial habitats of adults. During this vulnerable period, the megalopae of many coastal brachyurans cling to floating debris and ‘raft’ into their desired habitats, thereby reducing the energetic costs of moving towards shore. Indeed, larval brachyurans are sometimes the most abundant organisms in rafting communities (Donlan and Nelson 2003). The same fronts and eddies that tend to concentrate plankton and floating debris, providing potential rafting opportunities, also attract larger marine animals such as sea turtles and pelagic sea snakes. Raft‐seeking larvae in these situations may inadvertently (or intentionally) colonize these large living hosts and live for some time as ‘epibionts’—the term given to organisms that live on the surface of another organism. In rare instances, like crabs of the genus *Planes*, the rafting/epibiont tendencies of megalopae can become the permanent lifestyle for adults (Pfaller 2016).

**FIGURE 1:**
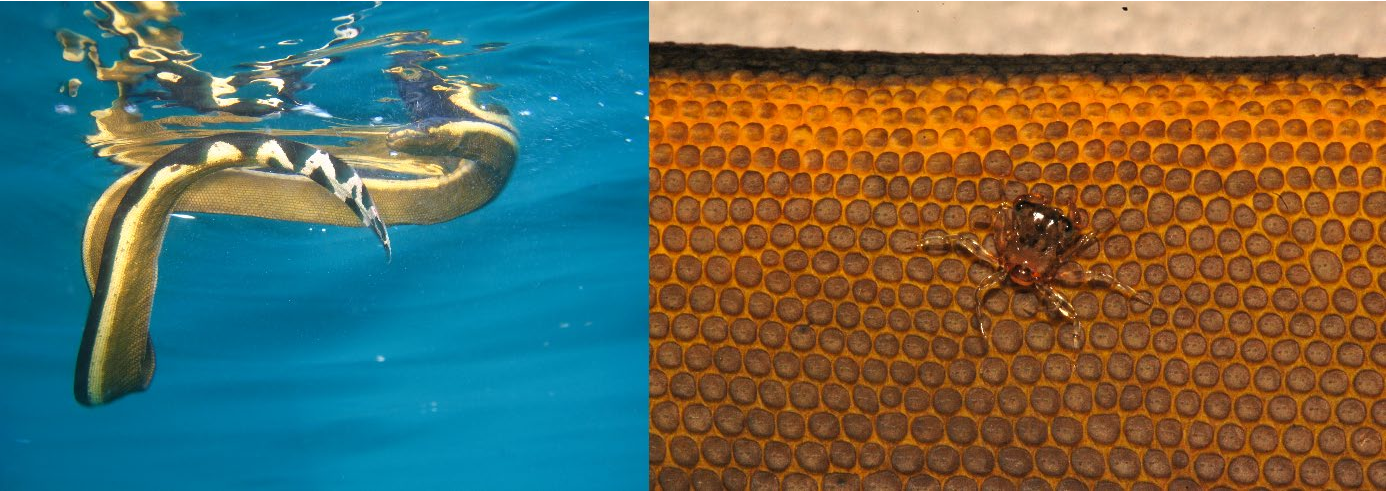
A specimen of *Hydrophis platurus* swimming along the surface and a grapsid megalopa clinging onto the snake's body (photo by J. Pfaller).

Pfaller et al. (2012) was the first to report decapod crustaceans as epibionts on sea snakes, specifically the yellow‐bellied sea snake, *Hydrophis platurus* (Linnaeus, 1766) (Figure 1). These snakes are pelagic “float‐and‐wait” predators, lying motionless at the surface of the open ocean waiting for small fish to seek refuge under their body before they strike (Brischoux and Lillywhite 2011). Of the 391 specimens of *H. platurus* sampled in Pacific Costa Rican waters by Pfaller et al. (2012), 74 snakes hosted a total of 157 decapod epibionts including two species of caridean shrimp (*Macrobrachium* sp. and *Atya* sp.), and juvenile individuals of *Planes minutus* (Linnaeus, 1758) (= *Planes major*) (*n* = 1) and *Achelous affinis* Faxon, 1893 (*n* = 5). The remaining epibionts included nine megalopae of *Plagusia squamosa* (Herbst, 1790) and 109 unidentified megalopae of the family Grapsidae. The authors hypothesized that the grapsid megalopae were likely *Planes minutus* for several reasons: (1) a juvenile *Planes minutus* was found on a sea snake during the study, (2) *Planes minutus* is an obligate rafting species and present in the area, where it frequently colonizes sea turtles, and (3) the other *Planes* species in the region, *Planes marinus* Rathbun, 1914, is less common and frequently found further offshore. However, because morphological descriptions of grapsid megalopae in the area were unavailable, the identity of these specimens remained undetermined.

The purpose of this study was to apply species‐level genetic tools to test whether the megalopae found riding on the sea snakes in Pfaller et al. (2012) were in fact *Planes minutus* . If the “*Planes* on a snake” hypothesis is confirmed, then it would indicate that snakes provide a temporary raft for *Planes* megalopae prior to settlement on flotsam or sea turtles. However, if the megalopae are other grapsid species, then it would suggest that rafting represents a successful strategy for larval recruitment in intra‐ and supratidal grapsids, but not other coastal brachyurans recruiting to the same habitats.

## 2 Methods

Specimen collection was outlined in Pfaller et al. (2012). Megalopae were collected during six research trips dedicated to studying the sea snakes in April, June, and October 2010 and March, May, and August 2011 (the latter includes one individual collected on July 31, 2011 and all others in early August 2011). When detected, megalopae were placed directly into vials of 95% ETOH and stored there until genetic analyses commenced in October 2022. Megalopae were identified using mitochondrial COI and 16 s rRNA sequences. All specimens were sequenced for COI to group them into MOTUs (Molecular Operational Taxonomic Units). Because none of the MOTUs matched any available species in the Barcode of Life Data System (BOLD) or NCBI's GenBank, 16S was sequenced from five representatives of each MOTU for further comparisons.

DNA was extracted from muscle tissue of one or more pereiopods per specimen using Chelex methods outlined in Appendix S1. The following primer sets were used for PCR amplification: jgLCO1490 and jgHCO2198 for COI (Geller et al. 2013); and CrustF1 (Costa et al. 2007) and CrustR2 (Matzen et al. 2011) for 16S. Amplifications were carried out in 25‐μl reaction volumes: 9.5 μL of water, 12.5 μL of GoTaq Green Master Mix (Promega, Madison, WI, USA), 1 μL of each 10 μM primer, and 1 μL of template DNA. PCR cycling parameters followed Lasley et al. (2023): initial denaturation at 95°C for 5 min; 4 cycles at 94°C for 30 s; 52°C (16S) or 50°C (COI) for 45 s; 72°C for 1 min; then 34 cycles at 94°C for 30 s; 47°C (16S) or 45°C (COI) for 45 s; and a final extension at 72°C for 8 min. PCR products were sent to TACGen (Richmond, CA, USA) for cycle sequencing. Resulting COI sequences were checked for stop codons using Geneious Prime 2025.2.1 (Biomatters Ltd.). GenBank accession numbers are listed in Table S1.

COI and 16S sequences were separately aligned using default settings of the MUSCLE Alignment tool in Geneious Prime. Neighbor joining (NJ), maximum likelihood (ML), and Bayesian inference (BI) phylogenetic trees were constructed using Geneious Tree Builder, and the RAxML and MrBayes plugins in Geneious Prime, respectively. *Armases ricordi* (H. Milne Edwards, 1853) was selected as an outgroup following Ip et al. (2015), using 16S and COI sequences of *A. ricordi* from Lasley et al. (2025). For the neighbor‐joining analysis, the genetic distance model selected was HKY and the analysis was conducted with 100 bootstraps. GTR GAMMA was selected as the nucleotide model for the maximum likelihood analysis, and the analysis was run for 100 bootstrap replicates. The following settings were selected for the Bayesian analysis: substitution model HKY85, rate variation gamma, gamma categories 4, chain length 1,100,000, subsampling frequency 200, heated chains 4, burn‐in length 100,000, and heated chain temp 0.2. Convergence of the runs was assessed using the Mr. Bayes plugin in Geneious Prime.

Species delimitation was inferred separately from COI and 16S data using Assemble Species by Automatic Partitioning (ASAP) (Puillandre et al. 2020), with the JC69 Jukes‐Cantor substitution model, and all other parameters set to default values. COI and 16S sequences from each delineated species were compared against sequences available in BOLD. The closest matches (> 99% identity) were downloaded, added to the alignment generated, and included in phylogenetic analyses.

## 3 Results

**FIGURE 2:**
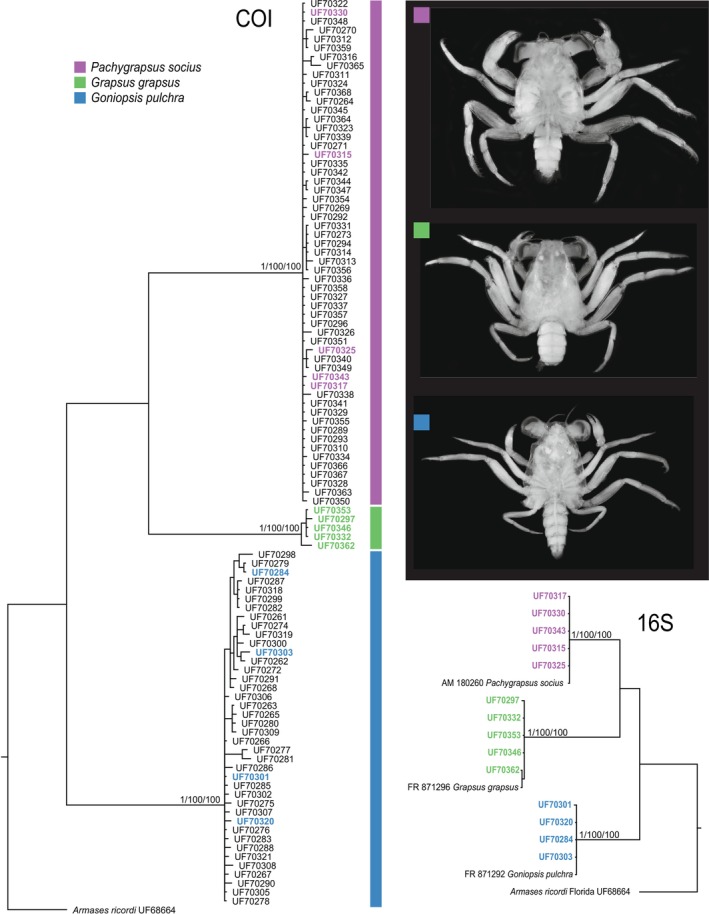
Phylogenetic relationships of megalopae collected from *H. platurus* based on mitochondrial COI (left) and 16S (bottom right) sequences. Trees show maximum‐likelihood topology with ML bootstrap values, NJ bootstrap values, and Bayesian posterior probabilities annotated at nodes.

Out of the 106 available specimens, COI sequences were obtained from 102, and 16S sequences from 14. Both COI and 16S phylogenies showed sequences to fall into three MOTUs that were also delineated by ASAP as three primary species hypotheses (Figure 2). BLAST searches for representatives of COI sequences returned matches with < 95% sequence identity. BLAST searches for the 16S sequences matched three species, *Goniopsis pulchra* (Lockington, 1877), *Grapsus grapsus* (Linnaeus, 1758), and *Pachygrapsus socius* Stimpson, 1871 with > 99% sequence identity. Forty of the 102 sequenced specimens matched *G. pulchra* , 5 matched *G. grapsus* , and 57 matched *P. socius* . The identification of matching GenBank sequences is considered reliable because some were published in taxonomic works by researchers with decades of taxonomic publications in grapsoid systematics (Schubart et al. 2005; Schubart 2011).

**FIGURE 3:**
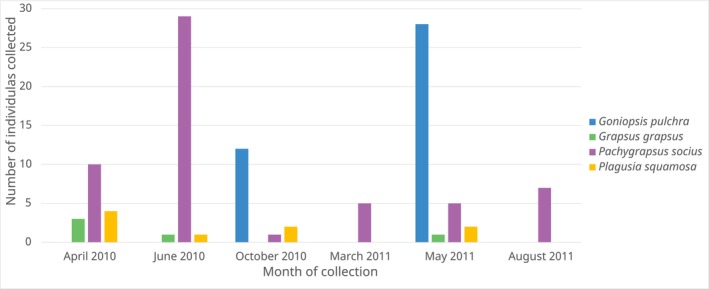
Number of larvae of each species found on *H. platurus* per month.

Thus, *Pachygrapsus socius* was the most common larval decapod epibiont of *H. platurus* (51.35%), followed by *Goniopsis pulchra* (36.04%), *Plagusia squamosa* (8.11%) and *Grapsus grapsus* (4.50%). Additionally, our results suggest that the megalopae of different grapsid species show some seasonality in their occurrence on *H. platurus* , likely reflecting the seasonality in their overall reproductive cycle (Figure 3). *G. pulchra* megalopae were found in May and October, *G. grapsus* in April through June, and *P. squamosa* in April through October. While *P. socius* megalopae were collected across all 6 months, they were the most abundant in June 2010.

Upon examination of the sequenced larvae, we noted that each MOTU corresponded to a distinct larval morphotype. *Grapsus grapsus* megalopae were distinguishable by their much larger body size, whereas *P. socius* and *G. pulchra* could be distinguished based on the presence of a rostral cleft and the shape of the eyes and chelae.

## 4 Discussion

The “*Planes* on a snake” hypothesis was rejected. None of the unidentified grapsid megalopae collected from *Hydrophis platurus* were *Planes minutus* or *Planes marinus* , both of which occur in the region (Chace 1951; Hendrickx 1995; Boschi 2000; Frick et al. 2011; Pfaller 2016). Aside from one juvenile *P. minutus* collected in April 2010, no other *Planes* crab (juvenile or megalopae) was found associated with *H. platurus* off Pacific Costa Rica.

Megalopae of another common rafting species, *Plagusia squamosa* , were documented on *H. platurus* by Pfaller et al. (2012) although in small numbers (*n* = 9). The presence of *Plagusia* but not *Planes* megalopae on *H. platurus* is perplexing. Both are known to be “rafting crabs” due to their pelagic lifestyle on flotsam or as epibionts on larger metazoan hosts, primarily sea turtles (Davenport 1992; Frick et al. 2004, 2011; Schubart and Ng 2000). It therefore came as no surprise that *Plagusia* megalopae were found on *H. platurus* , but why not *Planes*?

Larval recruitment of megalopae is unpredictable and mediated by many factors such as the date of release of larvae, ocean currents, lunar and diurnal cycles, and phylogenetic origin (Aiken and Waddy 1986; Hartnoll and Clark 2006; Milton et al. 2014; Moser and Macintosh 2001; Tilburg et al. 2008). The absence of *Planes* could be due to sampling bias if *Planes* megalopae were only recruiting during unsampled months. This, however, seems unlikely because: (1) ovigerous females rafting on flotsam or turtles likely release larvae constantly, as they are less constrained by seasonality than intertidal species are; (2) *Plagusia* megalopae were reported in four of 6 months of sampling, although in small numbers; and (3) there is no reason to think that currents would exclude *Planes* megalopae and not *Plagusia*. Lack of *Planes* megalopae could also be due to a relatively low regional abundance, or they might only recruit on flotsam well offshore.

Despite sharing a proclivity for rafting, the ecologies of *Planes* and *Plagusia* are distinct. *Plagusia* has been reported on sea turtles in the Eastern Tropical Pacific (Frick et al. 2011), but it is more commonly associated with flotsam and rocky intertidal habitats (Schubart and Ng 2000: material examined). Reports of *Planes*, however, are generally restricted to flotsam, such as *Sargassum* seaweed, jetsam, and sea turtles (Davenport 1992; Frick et al. 2004, 2011; Pfaller et al. 2014). The obligatory rafting lifestyle of *Planes* may make them more selective and therefore averse to settling on a suboptimal substrate, while the more generalist habitat preferences of *Plagusia* may not preclude such an inauspicious host as a sea snake. While *H. platurus* strongly avoids shoreward movements and will become incapacitated if cast ashore, the driftlines that snakes were collected were relatively close to shore (< 5 km), making these hosts even less suitable for *Planes* relative to *Plagusia*.

Aside from the absence of *Planes minutus* , the identity of the three species of non‐rafting crabs is also puzzling. *Goniopsis pulchra*, *Grapsus grapsus* , and *Pachygrapsus socius* are all inter‐ to supratidal species of crabs and represent almost the entire grapsid fauna in the study area, excluding *Geograpsus lividus* (H. Milne Edwards, 1837), an uncommon supratidal species (Abele and Kim 1989; Boschi 2000; Brusca 1980; Garth 1965, 1992; Hendrickx 1995; Poupin et al. 2005; Schubart et al. 2005; Vargas and Wehrtmann 2009). However, there are numerous other inter‐ to supratidal species of shore crabs in the area with megalopae that might be inclined to hitch rides on snakes—e.g., additional local species of grapsoid and related ocypodoid crabs of the families Plagusiidae, Macrophthalmidae, Sesarmidae, Gecarcinidae, Ocypodidae, and Varunidae. Several species of the latter two families are also known to raft on objects such as seaweed, wood, or plastic, but not metazoans. (Donlan and Nelson 2003; Goldstein et al. 2014; Thiel and Gutow 2005). The presence of almost the entire regional grapsid fauna, to the exclusion of the other shore crab fauna except the rafting species *Plagusia squamosa* , that belongs to the same superfamily, Grapsoidea, may indicate that rafting on snakes is a lineage‐specific behavioral adaptation. If this behavior was present in an ancestral grapsid, then hitching rides on diverse Metazoa—e.g., snakes and turtles—may have provided the mechanism for *Planes* to evolve from a typically inter‐ or supratidal grapsid lifestyle into a rather specialized hitch hiker.

## Author Contributions

**Soma Elefánti:** conceptualization (equal), data curation (equal), investigation (equal), visualization (supporting), writing – original draft (equal), writing – review and editing (equal). **Robert Lasley Jr:** conceptualization (equal), data curation (equal), formal analysis (equal), funding acquisition (equal), investigation (equal), methodology (equal), project administration (equal), supervision (equal), visualization (lead), writing – original draft (equal), writing – review and editing (equal). **Gustav Paulay:** conceptualization (equal), resources (equal), supervision (equal), writing – review and editing (equal). **Joseph B. Pfaller:** conceptualization (equal), data curation (equal), writing – review and editing (equal).

## Funding

This work was supported by the National Science Foundation, GECCO 1457769.

## Conflicts of Interest

The authors declare no conflicts of interest.

## Supporting information


**Table S1:** GenBank accession numbers of COI and 16S sequences of grapsid megalopae.
**Appendix S1:** Chelex Protocol used for extractions.

## Data Availability

All data supporting this study have been deposited in publicly accessible repositories. NCBI GenBank accession numbers for COI and 16S sequences will be made publicly available following manuscript acceptance. All alignments and ASAP species‐delimitation outputs are available in the Dryad Digital Repository under the DOI: https://doi.org/10.5061/dryad.5tb2rbphx.
