# Supplementary material for: Planes on a Snake? On the Identities of Crab Larvae Rafting on Sea Snakes
Source: Ecol Evol. 2026 May 31;16(6):e73742. doi: 10.1002/ece3.73742 (PMC13240343; doi:10.1002/ece3.73742)
Supplement: Supplementary file 1 — Table S1: GenBank accession numbers of COI and 16S sequences of grapsid megalopae. Appendix S1: Chelex Protocol used for extractions. [file ECE3-16-e73742-s001.zip › ece373742-sup-0001-TableS1.docx]

**Supplemental Tables**

Table S1. GenBank accession numbers of COI and 16S sequences of grapsid megalopae

| **Collection number** | **Field number** | **Species** | **COI** | **16S** |
| --- | --- | --- | --- | --- |
| UF070261 | JBP373 | *Goniopsis pulchra* | PX558156 | - |
| UF070262 | JBP374 | *Goniopsis pulchra* | PX558221 | - |
| UF070263 | JBP375 | *Goniopsis pulchra* | PX558162 | - |
| UF070264 | JBP376 | *Pachygrapsus socius* | PX558174 | - |
| UF070265 | JBP377 | *Goniopsis pulchra* | PX558178 | - |
| UF070266 | JBP378 | *Goniopsis pulchra* | PX558166 | - |
| UF070267 | JBP379 | *Goniopsis pulchra* | PX558222 | - |
| UF070268 | JBP380 | *Goniopsis pulchra* | PX558217 | - |
| UF070269 | JBP377 | *Pachygrapsus socius* | PX558179 | - |
| UF070270 | JBP383 | *Pachygrapsus socius* | PX558154 | - |
| UF070271 | JBP362 | *Pachygrapsus socius* | PX558167 | - |
| UF070272 | JBP363 | *Goniopsis pulchra* | PX558180 | - |
| UF070273 | JBP365 | *Pachygrapsus socius* | PX558199 | - |
| UF070274 | JBP366 | *Goniopsis pulchra* | PX558181 | - |
| UF070275 | JBP367 | *Goniopsis pulchra* | PX558185 | - |
| UF070276 | JBP368 | *Goniopsis pulchra* | PX558200 | - |
| UF070277 | JBP369 | *Goniopsis pulchra* | PX558168 | - |
| UF070278 | JBP370 | *Goniopsis pulchra* | PX558228 | - |
| UF070279 | JBP371 | *Goniopsis pulchra* | PX558190 | - |
| UF070280 | JBP372 | *Goniopsis pulchra* | PX558231 | - |
| UF070281 | JBP352 | *Goniopsis pulchra* | PX558223 | - |
| UF070282 | JBP353 | *Goniopsis pulchra* | PX558232 | - |
| UF070283 | JBP354 | *Goniopsis pulchra* | PX558201 | - |
| UF070284 | JBP355 | *Goniopsis pulchra* | PX558196 | PX558131 |
| UF070285 | JBP356 | *Goniopsis pulchra* | PX558182 | - |
| UF070286 | JBP357 | *Goniopsis pulchra* | PX558169 | - |
| UF070287 | JBP358 | *Goniopsis pulchra* | PX558160 | - |
| UF070288 | JBP359 | *Goniopsis pulchra* | PX558203 | - |
| UF070289 | JBP307 | *Pachygrapsus socius* | PX558233 | - |
| UF070290 | JBP360 | *Goniopsis pulchra* | PX558224 | - |
| UF070291 | JBP361 | *Goniopsis pulchra* | PX558202 | - |
| UF070292 | JBP304 | *Pachygrapsus socius* | PX558183 | - |
| UF070293 | JBP305 | *Pachygrapsus socius* | PX558234 | - |
| UF070294 | JBP308 | *Pachygrapsus socius* | PX558206 | - |
| UF070296 | JBP347 | *Pachygrapsus socius* | PX558204 | - |
| UF070297 | JBP348 | *Grapsus grapsus* | PX558211 | PX558138 |
| UF070298 | JBP349 | *Goniopsis pulchra* | PX558152 | - |
| UF070299 | JBP350 | *Goniopsis pulchra* | PX558225 | - |
| UF070300 | JBP351 | *Goniopsis pulchra* | PX558191 | - |
| UF070301 | JBP180 | *Goniopsis pulchra* | PX558170 | PX558129 |
| UF070302 | JBP181 | *Goniopsis pulchra* | PX558184 | - |
| UF070303 | JBP182 | *Goniopsis pulchra* | PX558205 | PX558132 |
| UF070305 | JBP184 | *Goniopsis pulchra* | PX558226 | - |
| UF070306 | JBP185 | *Goniopsis pulchra* | PX558157 | - |
| UF070307 | JBP186 | *Goniopsis pulchra* | PX558186 | - |
| UF070308 | JBP187 | *Goniopsis pulchra* | PX558212 | - |
| UF070309 | JBP188 | *Goniopsis pulchra* | PX558235 | - |
| UF070310 | JBP303 | *Pachygrapsus socius* | PX558236 | - |
| UF070311 | JBP133 | *Pachygrapsus socius* | PX558158 | - |
| UF070312 | JBP124 | *Pachygrapsus socius* | PX558207 | - |
| UF070313 | JBP134 | *Pachygrapsus socius* | PX558237 | - |
| UF070314 | JBP123 | *Pachygrapsus socius* | PX558213 | - |
| UF070315 | JBP136 | *Pachygrapsus socius* | PX558171 | PX558136 |
| UF070316 | JBP137 | *Pachygrapsus socius* | PX558155 | - |
| UF070317 | JBP145 | *Pachygrapsus socius* | PX558218 | PX558133 |
| UF070318 | JBP176 | *Goniopsis pulchra* | PX558214 | - |
| UF070319 | JBP177 | *Goniopsis pulchra* | PX558244 | - |
| UF070320 | JBP178 | *Goniopsis pulchra* | PX558192 | PX558130 |
| UF070321 | JBP179 | *Goniopsis pulchra* | PX558208 | - |
| UF070322 | JBP127 | *Pachygrapsus socius* | PX558150 | - |
| UF070323 | JBP125 | *Pachygrapsus socius* | PX558193 | - |
| UF070324 | JBP126 | *Pachygrapsus socius* | PX558159 | - |
| UF070325 | JBP135 | *Pachygrapsus socius* | PX558215 | PX558137 |
| UF070326 | JBP128 | *Pachygrapsus socius* | PX558209 | - |
| UF070327 | JBP129 | *Pachygrapsus socius* | PX558194 | - |
| UF070328 | JBP130 | *Pachygrapsus socius* | PX558247 | - |
| UF070329 | JBP131 | *Pachygrapsus socius* | PX558227 | - |
| UF070330 | JBP132 | *Pachygrapsus socius* | PX558151 | PX558134 |
| UF070331 | JBP110 | *Pachygrapsus socius* | PX558187 | - |
| UF070332 | JBP111 | *Grapsus grapsus* | PX558251 | PX558139 |
| UF070334 | JBP113 | *Pachygrapsus socius* | PX558241 | - |
| UF070335 | JBP117 | *Pachygrapsus socius* | PX558172 | - |
| UF070336 | JBP118 | *Pachygrapsus socius* | PX558188 | - |
| UF070337 | JBP119 | *Pachygrapsus socius* | PX558195 | - |
| UF070338 | JBP120 | *Pachygrapsus socius* | PX558219 | - |
| UF070339 | JBP121 | *Pachygrapsus socius* | PX558238 | - |
| UF070340 | JBP098 | *Pachygrapsus socius* | PX558229 | - |
| UF070341 | JBP099 | *Pachygrapsus socius* | PX558220 | - |
| UF070342 | JBP104 | *Pachygrapsus socius* | PX558173 | - |
| UF070343 | JBP106 | *Pachygrapsus socius* | PX558216 | PX558135 |
| UF070344 | JBP107 | *Pachygrapsus socius* | PX558175 | - |
| UF070345 | JBP108 | *Pachygrapsus socius* | PX558163 | - |
| UF070346 | JBP100 | *Grapsus grapsus* | PX558240 | PX558141 |
| UF070347 | JBP103 | *Pachygrapsus socius* | PX558176 | - |
| UF070348 | JBP105 | *Pachygrapsus socius* | PX558153 | - |
| UF070349 | JBP109 | *Pachygrapsus socius* | PX558248 | - |
| UF070350 | JBP122 | *Pachygrapsus socius* | PX558250 | - |
| UF070351 | JBP085 | *Pachygrapsus socius* | PX558210 | - |
| UF070353 | JBP089 | *Grapsus grapsus* | PX558164 | PX558140 |
| UF070354 | JBP090 | *Pachygrapsus socius* | PX558177 | - |
| UF070355 | JBP093 | *Pachygrapsus socius* | PX558230 | - |
| UF070356 | JBP094 | *Pachygrapsus socius* | PX558243 | - |
| UF070357 | JBP095 | *Pachygrapsus socius* | PX558197 | - |
| UF070358 | JBP096 | *Pachygrapsus socius* | PX558189 | - |
| UF070359 | JBP097 | *Pachygrapsus socius* | PX558246 | - |
| UF070362 | JBP088 | *Grapsus grapsus* | PX558239 | PX558142 |
| UF070363 | JBP400 | *Pachygrapsus socius* | PX558249 | - |
| UF070364 | JBP394 | *Pachygrapsus socius* | PX558165 | - |
| UF070365 | JBP392 | *Pachygrapsus socius* | PX558198 | - |
| UF070366 | JBP388 | *Pachygrapsus socius* | PX558242 | - |
| UF070367 | JBP387 | *Pachygrapsus socius* | PX558245 | - |
| UF070368 | JBP386 | *Pachygrapsus socius* | PX558161 | - |
