# Supplementary material for: Planes on a Snake? On the Identities of Crab Larvae Rafting on Sea Snakes
Source: Ecol Evol. 2026 May 31;16(6):e73742. doi: 10.1002/ece3.73742 (PMC13240343; doi:10.1002/ece3.73742)
Supplement: Supplementary file 1 — Table S1: GenBank accession numbers of COI and 16S sequences of grapsid megalopae. Appendix S1: Chelex Protocol used for extractions. [file ECE3-16-e73742-s001.zip › ece373742-sup-0002-AppendixS1.docx]

**Appendix S1**

**Supplemental Information**

**Chelex Protocol used for extractions:**

**Preparation of Chelex Mixture:**

1. With Bleach, sterilize a dry reagent spatula, and a small magnetic stir bar.
2. Prepare a 5-10% by weight slurry of Chelex 100 Resin (Biorad part 143- 3832,100-200 mesh Chelex, sodium form) and UV sterilized HPLC water. The most effective way to do this is to take a 50 ml sterile falcon tube, place in on a scale inside a small beaker and zero the scale. Then add 5 grams of Chelex and fill to 50ml mark with water. Precision is not critical. Sterile technique is.
3. Place sterile stirbar in tube and place on magnetic stirrer. Chelex settles quickly so if the slurry is not well mixed, your concentrations and results will be variable. Keeping the slurry well mixed, aliquot 300-500micro liters into 0.6 or 1.6ml eppendorf tubes (again, sterile) and cap immediately. If you have access to a laminar flow hood, that is a good place to do all of this. You may want to wipe down the scale and stirrer with a 10% bleach solution and/or UV sterilize prior to use.

**The Extraction:**

1. Turn on heating block. Set to 95°C. Fill holes with water.
2. Using sterile forceps (Flame over alcohol burner several times to sterilize), remove a small piece of tissue from your sample. This piece of tissue should be big enough to be visible, but not so big as to be easily visible. Imagine cutting a 0.2mm section of a standard staple. This is plenty big. Too much tissue may inhibit your reactions. Sterilize forceps between samples.
3. Vortex sample and chelex slurry for 10-15 seconds.
4. Spin samples briefly at high speed in a micro centrifuge
5. Incubate samples for 20 minutes at 95°C
6. Vortex samples again for 10-15 seconds (Be careful as steam may pop lid off of centrifuge tube. Hold lids down).
7. Spin tubes again at high speed in microcentrifuge.
8. Samples are ready to use. ONLY USE SUPERNATE FOR PCR REACTIONS. CHELEX BEAD WILL INACTIVATE TAQ!
